# Students’ attitudes toward digital learning during the COVID-19 pandemic: a survey conducted following an online course in gynecology and obstetrics

**DOI:** 10.1007/s00404-021-06131-6

**Published:** 2021-08-05

**Authors:** Gregor Leonhard Olmes, Julia Sarah Maria Zimmermann, Lisa Stotz, Ferenc Zoltan Takacs, Amr Hamza, Marc Philipp Radosa, Sebastian Findeklee, Erich-Franz Solomayer, Julia Caroline Radosa

**Affiliations:** 1 Department of Gynecology, Obstetrics and Reproductive Medicine, Saarland University Hospital, HomburgSaar, Germany; 2 Department of Gynecology and Obstetrics, Hospital Bremen-Nord, Bremen, Germany

**Keywords:** Digital learning, Gynecology and obstetrics, Corona virus, Medical education, Online teaching

## Abstract

**Purpose:**

The purpose of this survey was to assess medical students’ opinions about online learning programs and their preferences for specific teaching formats during COVID 19 pandemic.

**Methods:**

Between May and July 2020, medical students who took an online gynecology and obstetrics course were asked to fill in a questionnaire anonymously. The questionnaire solicited their opinions about the course, the teaching formats used (online lectures, video tutorials featuring real patient scenarios, and online practical skills training), and digital learning in general.

**Results:**

Of 103 students, 98 (95%) submitted questionnaires that were included in the analysis. 84 (86%) students had no problem with the online course and 70 (72%) desired more online teaching in the future. 37 (38%) respondents preferred online to traditional lectures. 72 (74%) students missed learning with real patients. All digital teaching formats received good and excellent ratings from > 80% of the students.

**Conclusion:**

The survey results show medical students’ broad acceptance of the online course during COVID 19 pandemic and indicates that digital learning options can partially replace conventional face-to-face teaching. For content taught by lecture, online teaching might be an alternative or complement to traditional education. However, bedside-teaching remains a key pillar of medical education.

## Introduction

The novel coronavirus (COVID-19) pandemic has posed challenges for medical education facilities, with the need to shift rapidly from the usual face-to-face teaching to online learning as routines in hospitals and medical schools have been interrupted by COVID-19 lockdown [1, 2]. Digital learning platforms like AMBOSS have been established in the past few years in German medical schools and universities and expand traditional teaching with digital options [3, 4]. Nevertheless, digital learning options provided by medical schools itself only took a minor part in students education due to the lack of infrastructure and technology at universities and negative attitudes among educators [5, 6]. Most digital learning studies conducted before the coronavirus outbreak had theoretical foci and demonstrated how such learning can improve students’ knowledge in domains such as communication skills [7, 8]. Data on medical students’ attitudes toward online education in clinical specialties, such as internal medicine, surgery, and gynecology, and techniques for the digital teaching of practical skills and examination administration, are sparse. The present survey-based study was conducted to assess medical students’ opinions about and attitudes toward online learning programs, and to identify their digital teaching format preferences.

## Materials and methods

### Setting and participants

The survey was conducted among medical students in the fifth clinical semester who completed a digital course between May and July 2020 at the Department of Gynecology, Obstetrics and Reproductive Medicine, Saarland University Hospital, Homburg/Saar, Germany. Regarding the level of familiarity with online teaching, students had access to the online learning platforms (e.g., AMBOSS) since 2018, but had no further experience with online teaching. The 5-day online course that took 3 h daily replaced a practical course in gynecology and obstetrics and was administered using different digital education formats: online lectures in gynecology and obstetrics, including live presentations on the main gynecological and obstetric diseases; video tutorials featuring real patient scenarios on topics such as vaginal delivery and caesarian section; and online education in gynecological examination and practical skills (e.g., cardiotocography).

After completing the course, students were invited to fill in a written questionnaire anonymously. The obtained questionnaires were stored at the hospital’s Department of Gynecology and Obstetrics. Questionnaire data and those on respondents’ gender and age, extracted from their course registration files, were entered into an SPSS (version 19; SPSS Inc., Chicago, IL, USA) database.

### Questionnaire

The survey questionnaire was used to investigate medical students’ opinions about digital learning in the context of the gynecological curriculum, and their attitudes toward different online teaching formats. It was adapted in 2020 for digital course evaluation from a standardized questionnaire developed by medical educators at our hospital for curriculum evaluation, which was approved in 2017.

The questionnaire had two parts. The first part comprised nine items covering students’ opinions about the online course and attitudes toward digital learning in general. Respondents rated their degree of agreement with the item statements (“true”; mostly true”; “neutral”; “mostly untrue”; “not true”). The second part was used to characterize students’ acceptance of three different digital teaching formats (lectures, videos featuring real patient scenarios, and online practical skills education). Rating options ranged on a five-item from (1) excellent, (2) good, (3) sufficient, (4) moderate, to (5) poor.

### Data analysis

Descriptive analysis was performed to characterize the data. Categorical variables are expressed as frequencies and percentages. Teaching format data were analyzed using the Chi-squared and Kruskal–Wallis tests. The analyses were performed using SPSS software (version 19; SPSS Inc., Chicago, IL, USA).

## Results

One hundred of 103 students who took the digital course submitted questionnaires after course completion (97% response rate). After the exclusion of two incomplete questionnaires, data from 98 (98%) questionnaires were included in the analysis. Forty (41%) of the respondents were male and 58 (59%) were female. The median age was 25 years (range, 23–50 years, Table 1).

**Table 1:** Characteristics of medical students (*n* = 98)

Characteristic	*n* (%) or median (range)
Gender	
Male	40 (41%)
Female	58 (59%)
Age (years)	25 (23–50)

### Students’ opinions about the online course content and structure

Most [*n* = 87 (89%)] students agreed that the online course topics were relevant for praxis [“true”, *n* = 58 (59%); “mostly true”, *n* = 29 (30%)]. Three (3%) students found the chosen topics not to be relevant [“mostly untrue”, *n* = 1 (1%); “not true”, *n* = 2 (2%)]. Seventy-four (76%) students agreed that the instruction of the online course was well prepared [“true”, *n* = 40 (41%); “mostly true”, *n* = 34 (35%)], whereas six (6%) students did not [“mostly untrue” and “not true”, *n* = 3 (3%) each]. A majority [*n* = 84 (86%)] of students could follow the online course [“true”, *n* = 53 (54%); “mostly true”, *n* = 31 (32%)]; four (4%) students indicated that they had difficulty following it [“mostly untrue”, *n* = 1 (1%); “not true”, *n* = 3 (3%)]. Eighty-four (86%) students agreed that the online course improved their knowledge of gynecology and obstetrics [“true”, *n* = 53 (54%); “mostly true”, *n* = 31 (32%)]. Eleven (11%) students had a neutral position on this statement, and three (3%) students disagreed with it (Table 2).

**Table 2:** Questionnaire responses (*n* = 98)

Item	*n* (%)
The topics chosen for the online course are relevant for praxis	
True	58 (59%)
Mostly true	29 (30%)
Neutral	8 (8%)
Mostly untrue	1 (1%)
Not true	2 (2%)
The instruction of the online course was well prepared	
True	40 (41%)
Mostly true	34 (35%)
Neutral	18 (18%)
Mostly untrue	3 (3%)
Not true	3 (3%)
I could follow the online course	
True	53 (54%)
Mostly true	31 (32%)
Neutral	10 (10%)
Mostly untrue	1 (1%)
Not true	3 (3%)
The online course improved my understanding of gynecology and obstetrics	
True	53 (54%)
Mostly true	31 (32%)
Neutral	11 (11%)
Mostly untrue	0 (0%)
Not true	3 (3%)
An online course can replace a practical course with patient contact in the hospital	
True	4 (4%)
Mostly true	9 (9%)
Neutral	11 (11%)
Mostly untrue	22 (23%)
Not true	52 (53%)
I would prefer an online lecture instead of a traditional lecture	
True	19 (19%)
Mostly true	18 (18%)
Neutral	15 (16%)
Mostly untrue	27 (28%)
Not true	19 (19%)
I am looking forward to the integration of more online teaching in the future	
True	45 (46%)
Mostly true	25 (26%)
Neutral	14 (14%)
Mostly untrue	5 (5%)
Not true	9 (9%)
I missed learning with patient contact for better understanding	
True	45 (46%)
Mostly true	27 (28%)
Neutral	13 (13%)
Mostly untrue	10 (10%)
Not true	3 (3%)
In the future, I wish to learn further with real patients	
True	72 (74%)
Mostly true	17 (17%)
Neutral	6 (6%)
Mostly untrue	1 (1%)
Not true	2 (2%)

### Acceptance of the three digital teaching formats

Overall, ratings for all three teaching formats were good or excellent. Forty-four (45%) rated the online lectures as excellent and 38 (39%) rated them as good [total, *n* = 82 (84%)]. Similar results were obtained for the video tutorials [*n* = 86 (88%); excellent, *n* = 47 (48%); good, *n* = 39 (40%); < good, *n* = 12 (12%)] and online education in practical skills [*n* = 87 (89%); excellent, *n* = 51 (52%); good, *n* = 36 (37%); < good, *n* = 11 (11%)] (Table 3). The median rating was highest for the online lectures [1.7 (range, 1–4)], followed by the video tutorials [1.7 (range, 1–5)] and online education in practical skills [1.6 (range, 1–6)], but the ratings did not differ significantly among formats (*p* = 0.368).

**Table 3:** Evaluation of the digital teaching formats

Evaluate every format with a grade	
Format	*n* (%)
Online lectures	
(1) Excellent	44 (45%)
(2) Good	38 (39%)
(3) Sufficient	13 (13%)
(4) Moderate	3 (3%)
(5) Poor	0 (0%)
Median (range)	1.7 (1–4)
Video tutorials featuring real patient scenarios	
(1) Excellent	47 (48%)
(2) Good	39 (40%)
(3) Sufficient	9 (9%)
(4) Moderate	1 (1%)
(5) Poor	2 (2%)
Median (range)	1.7 (1–5)
Online education in gynecological examination and practical skills	
(1) Excellent	51 (52%)
(2) Good	36 (37%)
(3) Sufficient	10 (10%)
(4) Moderate	0 (0%)
(5) Poor	1 (1%)
Median (range)	1.6 (1–6)

### Students’ opinions about online learning

Most [*n* = 74 (76%)] students indicated that an online course cannot replace a practical course with patient contact in the hospital. Eleven (13%) students had neutral responses to this item and 13 (13%) students agreed that an online course can replace a practical course [“true”, *n* = 4 (4%); “mostly true”, *n* = 9 (9%)]. 47 students (46%) indicated that they did not prefer online to traditional lectures [“mostly untrue”, *n* = 27 (28%); “not true”, *n* = 19 (19%)]; 38% (*n* = 37) of students preferred online lectures [“true”, *n* = 19 (19%); “mostly true”, *n* = 18 (18%)] and 15% (*n* = 16) had neutral opinions on this statement. Seventy (71%) students indicated that they would welcome the integration of more online teaching in the future [“true”, *n* = 45 (46%); “mostly true”, *n* = 25 (26%)] (Table 2).

### Students’ opinions about learning without real patients

Most [*n* = 72 (74%)] students missed learning with real patients [“true”, *n* = 45 (46%); “mostly true”, *n* = 27 (28%)]; 13 (13%) students had a neutral opinion on this statement and 13 (13%) students did not agree with it [“mostly untrue”, *n* = 10 (10%); “not true”, *n* = 3 (3%)]. The majority [*n* = 89 (91%)] of students indicated that they desired further learning with real patients [“true”, *n* = 72 (74%); “mostly true”, *n* = 17 (17%)] (Table 2).

## Discussion

This survey of medical students who took an online course in gynecology and obstetrics that replaced a face-to-face practical course emphasizes that for medical students online teaching can be a good option for learning during COVID 19 pandemic, but cannot replace conventional teaching in the university hospitals. Most participants indicated that they desire more integration of online teaching in the future. In addition, all three digital teaching formats used in the course (online lectures, video tutorials featuring real patient scenarios and online education in practical skills) received good ratings.

As modern technology and web-based systems are now integral parts of everyday life, medical students’ demand for and use of digital learning options (e.g., self-directed programs from local universities and other institutions) has increased [9]. These students’ use of social media platforms has also increased and provides the opportunity to interact, discuss cases, and study in session for examinations [10]. The COVID-19 pandemic obligated students to engage in digital learning and revitalized digital learning options [11]. For example, during the period of campus closure, universities in China implemented 24,000 online courses (401 including virtual experimental simulation and 22 providing learning platforms), coordinated by the Chinese Ministry of Education, which monitored online education progress and quality [12]. A survey of 39,854 students at the Southeast University in China emphasized the importance of this tremendous undertaking; 50% of respondents believed that the planned teaching objectives were attained fully [12]. The survey also revealed that students prefer a mixture of different learning formats, such as the use of recorded videos in combination with live courses; this mixed teaching style appeared to increase students’ participation and mitigate the impact of unstable networks [12]. The researchers concluded that in the absence of face-to-face communication, teachers need to put greater effort into preparing for online courses, innovating and designing lessons [12]. The good ratings that students gave to the different teaching formats in this study are in line with these findings, but our students also indicated that teaching and learning with real patients is elementary for future curricula.

Data from the University of Washington document a tenfold increase in digital education in pathological anatomy for distant students during the COVID-19 pandemic. Such a course similar to ours was a comprehensive 2-week remote-learning program for third- and fourth-year students that included lectures, slide presentations, virtual discussion groups, and case-based activities [13]. A survey on course effectiveness yielded positive student feedback; in contrast to our findings, the students found the educational quality to be equivalent to that of an in-person course [13]. This difference might be explained by the differences in course objectives and teaching subjects, which included a practical skill development in our course in comparison to a theoretical subject like pathological anatomy in the other.

Schulz-Quach et al. examined 670 medical students’ acceptance of a digital course in palliative care that included online lectures, YouTube videos, self-reflection exercises, and interactive case management, and culminated in a multiple-choice examination. In line with our results, responses to a standardized university questionnaire on students’ palliative care competence revealed broad acceptance of digital learning in e-learning and non-e-learning subgroups [14]. The authors also observed no difference in self-estimated palliative care competence between groups, argued to be attributable to the lack of experience-based learning with real patients. Like our findings, this result emphasizes the need for patient contact in the teaching of skills to medical students.

Investigations in 205 medical students found that video lectures were as effective as live lectures for preparation for a medical examination and could be complementary in medical education. The majority, 48% of students preferred live lectures and 27% preferred video lectures [15]. These results are in line with our findings of lecture rating and some students’ preference of online over traditional lectures.

The affinity toward digital learning is known to vary among students; based on a two-semester-long prospective study, Backhaus et al. identified a digital native and traditional learner type. Digital learners had greater difficulty with traditional lectures than with e-learning, whereas traditional learners showed no difference in knowledge gain between formats. The author emphasized that medical educators should be aware of changes in learning habits, and our results may provide hints of such changes [16].

The implementation of video tutorials can be part of skills training like ultrasound teaching, improving participants knowledge and hands-on skills [17]. In a randomized study, Gonzalves et al. investigated the use of video tutorials and slide shows to teach maneuvers for shoulder dystocia to medical and midwifery students. At the end of the course students were evaluated by graders. Scores in practice and theory were significantly higher among students in the video tutorial group, leading the authors to conclude that video tutorials improved learning relative to standard lectures alone [18]. Our survey also showed broad acceptance of video tutorials among medical students.

Schlupeck et al. reported medical students’ acceptance of an online course on wound care; 69% of students found the online course to be superior to a conventional lecture, and students’ perceived competence increased significantly. In line with these results, our students rated the online course highly [19].

Despite the good performance of the digital teaching formats and the broad acceptance of digital learning, our results reflect an important limitation of digital education, namely that it does not allow for learning with real patients. Our students indicated that the online course could not replace a practical course with patient contact and indicated that they missed patient-centered learning, an elementary educational component that enhances students’ understanding and awareness of the complexity of patient care [20, 21]. In addition to bedside-teaching, practical units on diverse topics (e.g., intrauterine devices, conization, laparoscopy, and obstetric ultrasound) with patient contact or realistic scenarios improve medical students’ knowledge and understanding [22–30].

A review of e-based learning programs for nursing students yielded that e-learning is a flexible teaching method, but is not superior to face-to-face patient simulation. The authors concluded that combined traditional and e-based learning could achieve the best results [31].

The limitations of this study include those inherent to survey-based research. Our survey reflects only one semester during COVID 19 pandemic and was performed as single center. Experiences from other universities would be interesting to compare for further approach. In addition, we evaluated only students’ opinions, and not the effectiveness of online teaching, as reflected for example in examination scores. Thus, although the students showed broad acceptance of online teaching, we cannot comment on the effectiveness of this educational approach in terms of knowledge and medical skill acquisition. As the degree of knowledge transfer is a key indicator in the evaluation of teaching methods, additional studies of the relative effectiveness of online and conventional teaching formats are needed.

## Conclusion

In this study, we found a high degree of acceptance of digital teaching formats among medical students during COVID 19 pandemic. In particular, the teaching of knowledge via lectures seems to be partially transferable to an online format. To learn practical skills and patient treatment, medical students require patient-centered education. These findings can be used to further implement digital online formats into students’ medical education.

## Data Availability

The dataset used and analyzed during the current study is available from the corresponding author on reasonable request.
